# Identification de cas de borréliose à tiques par qPCR à partir de tests rapides de diagnostic du paludisme lors du Grand Magai de Touba au Sénégal

**DOI:** 10.48327/mtsi.v5i4.2025.732

**Published:** 2025-08-16

**Authors:** Coumba DIOUF, Ihssane OUADDANE, Georges DIATTA, Mamadou Lamine Bara GOUMBALA, Abdourahmane SOW, Déguène FAM, Mbayang FAYE, Philippe GAUTRET, Cheikh SOKHNA

**Affiliations:** 1 Aix Marseille Université, Institut de recherche pour le développement (IRD), Assistance publique-Hôpitaux de Marseille (AP-HM), Service de santé des armées (SSA), Maladies infectieuses négligées et émergentes au Sud (MINES), Dakar, Sénégal; 2 Aix Marseille Université, IRD, AP-HM, SSA, RITMES, Marseille, France; 3 Institut hospitalo-universitaire (IHU)-Méditerranée Infection, Marseille, France; 4 IRD, Université Cheikh Anta Diop (UCAD), MINES, Dakar, Sénégal Auteur

**Keywords:** Fièvre, Borréliose, Paludisme, Arboviroses, Tiques, TDR, Rassemblement de masse, Mbacké, Touba Keur Niang, Darou Tanzil, Ndindy, Serigne Saliou, Daroul Manane, Bagdad, Touba, Sénégal, Afrique subsaharienne, Fever, Borreliosis, Malaria, Arboviruses, Ticks, RDT, Mass gathering, Mbacke, Touba Keur Niang, Darou Tanzil, Ndindy, Serigne Saliou, Daroul Manane, Bagdad, Touba, Senegal, Sub-Saharan Africa

## Abstract

**Introduction:**

Les tests de diagnostic rapide (TDR) antigéniques ont été développés pour faciliter le diagnostic du paludisme dans les zones d’endémie palustre. Le matériel sanguin collecté pour ces tests peut être utilisé pour le diagnostic d’autres infections.

**Méthode:**

Une extraction d’acides nucléiques suivie d’une identification par PCR du virus de la dengue (DENV), *Borrelia* spp, *Bartonella* spp et *Coxiella burnetti* a été effectuée sur des TDR collectés dans différents structures sanitaires de Touba lors du Grand Magal de Touba (GMT), un rassemblement de masse connu pour ses risques infectieux.

**Résultats:**

2 381 TDR ont été collectés dans huit structures sanitaires du département de Mbacké en 2022 et 2023 pendant le GMT. Treize cas (0,5%) de borréliose de type fièvre récurrente transmise par les tiques ont été identifiés par qPCR dont deux cas de co-infection paludisme-borréliose. Aucun des échantillons n’a été positif pour le virus de la DENV, *Bartonella* spp et *C. burnetti.*

**Conclusion:**

Nos résultats confirment que les antigènes de TDR peuvent être exploités pour le diagnostic de fièvres non palustres comme la borréliose présente chez certains participants fébriles du GMT. Les TDR du paludisme, utilisés sur le terrain, constituent une source facilement accessible d’échantillons cliniques pour étudier l’épidémiologie des causes de fièvre d’origine inconnue dans le contexte du GMT.

## Abstract (en)

**Introduction:**

Antigen rapid diagnostic tests (RDTs) have been developed to facilitate malaria diagnosis in endemic areas. The blood samples collected for these tests can also be used to diagnose other infections.

**Methods:**

We performed nucleic acid extraction followed by PCR identification of dengue virus (DENV), *Borrelia* spp., *Bartonella* spp., and *Coxiella burnetti* on RDTs collected from different health facilities in Touba during the Grand Magal of Touba (GMT), a mass gathering known for its infectious disease risks.

**Results:**

A total of 2,381 RDTs were collected from eight healthcare facilities in the Mbacke department in 2022 and 2023 during the GMT. Thirteen cases (0.5%) of tick-borne relapsing fever borreliosis were identified by quantitative PCR (qPCR), including two cases of malaria-borreliosis co-infection. None of the samples tested positive for DENV, *Bartonella* spp., or *C. burnetti.*

**Conclusion:**

Our results confirm that RDT antigens can diagnose non-malaria fevers, such as borreliosis, in some GMT participants with fever. Malaria RDTs used in the field are an easily accessible source of clinical samples for studying the epidemiology of fevers of unknown origin in GMT contexts.

## Introduction

Le Grand Magal de Touba (GMT) est un événement religieux organisé par la communauté mouride, une confrérie musulmane du Sénégal, qui a lieu le 18 Safar de chaque année selon le calendrier musulman. L’événement commémore le départ en exil au Gabon de Cheikh Ahmadou Bamba Mbacké, fondateur de la confrérie, en 1895. Il attire entre 4 et 5 millions de pèlerins, ce qui en fait le plus grand rassemblement de masse en Afrique de l’Ouest. Cet événement présente un risque majeur de transmission de maladies infectieuses, dont la propagation peut être favorisée par la densité de population des participants dans les sites les plus fréquentés et l’origine géographique très large des pèlerins [10]. Une étude menée pendant le GMT en 2016 a montré que, parmi 20 850 patients, les maux de tête étaient les symptômes les plus fréquents (28,2%), suivis par les symptômes gastro-intestinaux (22%), la fièvre (17,2%), les symptômes respiratoires (17,1%) et le paludisme, qui touchait 2,4% des patients [11]. De même, une étude menée de 2018 à 2021 au centre de santé de Mbacké pendant le GMT a montré que sur un total de 141 patients, 45 (31,9%) ont été testés positifs pour au moins un pathogène sanguin, dont 21,3% pour *Plasmodium falciparum,* 5,7% pour *Borrelia* spp. et 5% pour le virus de la dengue [5]. Parmi 204 patients fébriles testés pour le paludisme avec des tests de diagnostic rapide du paludisme (TDR), 41 (20,1%) se sont révélés positifs. Ces TDR ont été réutilisés pour rechercher des pathogènes non palustres dont la borréliose par séquençage. Les résultats ont montré un total de 18 cas (8,8%) de borréliose dont 8,3% chez les patients non paludéens et 0,5% chez les patients atteints du paludisme [7]. Cette étude vise à identifier, par PCR, certains pathogènes possiblement responsables des cas de fièvre non palustre à l’aide du matériel antigénique extrait des cassettes de TDR lors des GMT de 2022 et 2023.

## Méthodes

Des TDR de marque ParaHIT (Akray Healthcare, Inde) et le Bioline^TM^ Malaria Ag P. f/Pan (Abbott, USA) détectant uniquement *P.falciparum* ont été collectés dans les structures sanitaires du département de Mbacké suivantes: Centres de santé de Touba Keur Niang, Darou Tanzil, Mbacké, Ndindy, Serigne Saliou, Daroul Manane et Postes de santé de Bagdad et Touba HLM. Après la collecte des échantillons, qui s’est déroulée du 13 au 17 septembre 2022 et du 2 au 6 septembre 2023, nous avons procédé à l’ouverture des TDR, soulevé la bandelette, gratté la membrane de nitrocellulose qui adhère complètement au support et l’avons transféré dans un tube Eppendorf de 1,5 ml. L’ADN et de l’ARN ont été extraits à l’aide de l’instrument KingFisher™ Flex. Tous les essais de PCR quantitative en temps réel ont été réalisés à l’aide d’un thermocycleur C1000 Touch™ (BioRad, Hercules, CA, USA) pour détecter *P. falciparum, P. malariae, P. vivax, P. ovale,* le virus de la dengue (DENV), *C. burnetti, Borrelia* spp. et *Bartonella* spp. (Tableau I). Les amplifications par PCR en temps réel ont également été réalisées à l’aide de la trousse LightCycler^®^ 480 Probes Master kit (Roche Diagnostics, France) conformément aux recommandations du fabricant. Des contrôles négatifs (mélange PCR) et positifs (ADN de souches bactériennes ou parasitaires ou ARN de souches virales) ont été inclus dans chaque série. Un seuil de cycle (CT) de 35 a été utilisé pour les résultats positifs après amplification.

**Tableau I:** Amorces et sondes utilisées pour l’amplification par qPCR des acides nucléiques provenant des tests TDR pour le paludisme

Gene cible	Nom de l’amorce	Séquence
IS 1111 / *Coxiella burnetii*	CB_IS1111_0706F	CAAGAAACGTATCGCTGTGGC
	CB_IS1111_0706R	CACAGAGCCACCGTATGAATC
	CB_IS1111_0706P	6FAM-CCGAGTTCGAAACAATGAGGGCTG
ITS2 / *Bartonella* spp.	Bart-ITS2_F	GGGGCCGTAGCTCAGCTG
	Bart-ITS2_R	TGAATATATCTTCTCTTCACAATTTC
	Bart-ITS2_P	FAM-CGATCCCGTCCGGCTCCACCA-TAMRA
16S ribosomal RNA / *Borrelia* spp.	Bor16S-F	AGCCTTTAAAGCTTCGCTTGTAG
	Bor16S-R	GCCTCCCGTAGGAGTCTGG
	Bor16S-P	FAM-CCGGCCTGAGAGGGTGAACGG-TAMRA
*Plasmodium falciparum*	Plasmo_18S_2_MBF	AGGCAACAACAGGT CTGTGA
	Plasmo_18S_2_MBR	GCAATAATCTATCCCCATCACG
	Plasmo_18S_2_MBP	6FAM- GAACTAGGCTGCACGCGTGCTACA-TAMRA
*Plasmodium malariae*	Plasmo-mal_F	TAGCATATATTAAAATTGTTGCAG
	Plasmo-mal_R	GTTATTCCATGCTGTAGTATTCA
	Pmal_P	6FAM- TGCATGGGAATTTTGTTACTTTGAGT - TAMRA
*Plasmodium ovale*	Plasmo-ova_F	TAGCATATATTAAAATTGTTGCAG
	Plasmo-ova_R	GTTATTCCATGCTGTAGTATTCA
	Pova_P	6FAM- TGCATTCCTTATGCAAAATGTGTTC - TAMRA
*Plasmodium vivax*	Plasmo-viva_F	TAGCATATATTAAAATTGTTGCAG
	Plasmo-viva_R	GTTATTCCATGCTGTAGTATTCA
	Pviva_P	6VIC- CGACTTTGTGCGCATTTTGC - TAMRA
Virus de la dengue	Denv_F	GGA CTA GAG GTT AGA GGA GAC CCC
	Denv_R	GAG ACA GCA GGA TCT CTG GTC
	Denv_P	6FAM-AGC ATA TTG ACG CTG GGA-MGB-BHQ1

## Résultats

Au total, 2 381 TDR du paludisme ont été collectés, dont 1 014 en 2022 et 1 367 en 2023. La lecture visuelle des tests antigéniques avait détecté 213 positifs pour *P.falciparum* (9%) dont 177 (17,5%) en 2022 et 36 (2,7%) en 2023. En qPCR, 194 échantillons (8,1%) étaient positifs pour *P. falciparum* (Tableau II), dont 138 (13,6%) en 2022 et 56 (4,1%) en 2023. Aucun des échantillons n’a été testé positif pour d’autres espèces de *Plasmodium.* Nous avons identifié, par qPCR, 13 cas de borréliose de type fièvre récurrente transmise par les tiques (TBRF) dont 1 cas en 2022 et 12 en 2023. Parmi ces 13 échantillons positifs pour *Borrelia,* 11 concernaient des TDR négatifs en lecture et négatifs en qPCR pour *P. falciparum*, 1 concernait un TDR négatif en lecture mais positif en qPCR pour *P. falciparum,* et 1 concernait un TDR positif en lecture et confirmé positif en qPCR pour *P. falciparum*. La prévalence globale de la borréliose dans les TDR négatifs était de 0,5%. Tous les échantillons étaient négatifs pour DENV, *C. burnetti* et *Bartonella* spp.

**Tableau II:** Prévalence de différents pathogènes identifiés par qPCR à partir du matériel génétique extrait des TDR dans diverses structures sanitaires du département de Mbacké, au Sénégal

Variables étudiées	Keur Niang	Darou Tanzil	Daroul Manane	Mbacké	Serigne Saliou	Ndindy (Centre de santé)	Bagdad	Touba HLM	Total
**2022**									
*Plasmodium falciparum*	95 (29,7%)	12 (5,4%)	5 (3,8%)	7 (5,7%)	14 (26,6%)	0	5 (7%)	0	138 (13,6%)
*Plasmodium ovale*	0	0	0	0	0	0	0	0	0
*Plasmodium vivax*	0	0	0	0	0	0	0	0	0
*Plasmodium malariae*	0	0	0	0	0	0	0	0	0
*Borrelia* spp.	0	0	0	1 (0,8%)	0	0	0	0	1 (0,1%)
*Bartonnella* spp.	0	0	0	0	0	0	0	0	0
*Coxellia Burnetti*	0	0	0	0	0	0	0	0	0
Virus de la dengue	0	0	0	0	0	0	0	0	0
**2023**									
*Plasmodium falciparum*	6 (4%)	22 (3,5%)	6 (7,5%)	3 (3,2%)	17 (6,4%)	1 (0,7%)	0	1 (14%)	56 (4,1%)
*Plasmodium ovale*	0	0	0	0	0	0	0	0	0
*Plasmodium vivax*	0	0	0	0	0	0	0	0	0
*Plasmodium malariae*	0	0	0	0	0	0	0	0	0
*Borrelia* spp.	0	3 (0,5%)	3 (3,8%)	0	4 (1,5%)	2 (1,3%)	0	0	12(0,9%)
*Bartonnella* spp.	0	0	0	0	0	0	0	0	0
*Coxellia Burnetti*	0	0	0	0	0	0	0	0	0
Virus de la dengue	0	0	0	0	0	0	0	0	0

## Discussion

Le paludisme causé par *P. falciparum, P. malariae et P. ovale* est une maladie endémique au Sénégal et toute la population est exposée à cette dernière [9]. Les TDR ont été développés pour la première fois dans les années 1990, facilitant le diagnostic du paludisme dans les zones d’endémie subsahariennes [6]. Ils présentent de nombreux avantages, tels que la rapidité d’obtention des résultats (5-20 minutes maximum), leur facilité d’interprétation et leur faible coût. En outre, les TDR du paludisme ont permis de prendre en charge rapidement les patients fébriles, en fournissant un diagnostic définitif du paludisme, de sorte que le traitement antipaludique peut être administré rapidement. La TBRF, due à des infections à *Borrelia* transmises par des tiques du genre *Ornithodoros,* est une cause majeure de maladie dans plusieurs régions d’Afrique [2]. La répartition géographique de la TBRF est liée à des climats plutôt secs [12].

Une étude menée au centre de santé de Mbacké en 2022 et 2023 a montré une prévalence de 0% et 8,6% respectivement pour la TBRF à *Borrelia* lorsqu’elle est détectée par qPCR dans le sang veineux total de patients fébriles (données non publiées). L’absence d’infection à *Borrelia* observée en 2023 dans les TDR collectés au centre de santé de Mbacké pourrait s’expliquer par le fait que la quantité de sang capillaire utilisée pour les TDR (environ 10 μl) est beaucoup plus faible que celle utilisée pour la qPCR (200 pl de sang veineux) pendant laquelle il y a une amplification qui permet de détecter des infections plus faibles. Ces résultats confirment que la TBRF peut être présente chez les patients fébriles au cours du GMT, ce qui justifie qu’elle soit recherchée chez les patients fébriles non paludéens, de préférence sur sang total.

Une épidémie de dengue a été diagnostiquée à Fatick le 15 septembre 2018 suivie de la détection d’un foyer à Touba où s’est tenu le GMT le 28 octobre 2018 [1]. Parmi 832 échantillons inclus dans une étude menée chez des patients fébriles dans différentes structures sanitaires de Touba en 2018,22,71% (189/832) ont été positifs au virus de la dengue par qPCR [3]. Des études menées pendant les GMT de 2018 et 2022 avaient montré respectivement 7 cas (26,9%) et 2 cas (5,8%) de dengue, détectée par qPCR dans le sang veineux, chez les patients consultant pour une fièvre au centre de santé de Mbacké [4,5]. Dans notre étude, aucun cas de dengue n’a été découvert, ce qui pourrait s’expliquer par la faible quantité de sang capillaire utilisé pour les TDR ou par l’absence du virus au moment du GMT.

Notre étude présente certaines limites. Tous les pathogènes pouvant causer des fièvres non palustres n’ont pas été recherchés. Les données cliniques, démographiques et l’origine géographique des patients n’ont pas été documentées du fait du protocole de l’étude. En outre, le long délai entre la réalisation des TDR et le diagnostic par PCR pourrait être raccourci à l’aide d’une logistique appropriée.

Les TDR du paludisme utilisés sur le terrain constituent néanmoins une source facilement accessible d’échantillons cliniques pour réaliser le diagnostic étiologique des fièvres et, éventuellement, étudier l’épidémiologie des causes de fièvre d’origine inconnue dans le contexte du GMT.

## Ethique

Le protocole a été approuvé par le Comité national d’éthique pour la recherche en santé au Sénégal (SEN17/62) et réalisé conformément aux bonnes pratiques cliniques recommandées par la déclaration d’Helsinki et ses amendements.

## Financement

Cette étude est soutenue par l’Institut hospitalo-universitaire (IHU) Méditerranée Infection, l’Agence nationale de la recherche française dans le cadre du programme des Investissements d’avenir, référence ANR-10-IAHU-03.

## Contribution des auteurs

C. D. a contribué à la conception expérimentale, a rédigé le manuscrit, a réalisé la technique qPCR. I. O. a contribué à la conception expérimentale. G. D. a collecté des échantillons et révisé le manuscrit. M. G. a réalisé la technique qPCR. A. S., D. F., M. F. ont réalisé la dissection des TDR. P. G. a rédigé le projet original, a contribué à la conception expérimentale, a révisé le manuscrit et a coordonné le travail. C. S. a rédigé le projet original, a contribué à la conception expérimentale et a coordonné le travail. Tous les auteurs ont contribué et approuvé la version actuelle du manuscrit.

## Conflits d’intérêts

Les auteurs ne déclarent aucun conflit d’intérêts.
