# Supplementary material for: Identification of tick-borne borreliosis cases by qPCR from rapid malaria diagnostic tests during the Grand Magal of Touba in Senegal
Source: Med Trop Sante Int. 2025 Aug 16;5(4):mtsi.v5i4.2025.732. [Article in French] doi: 10.48327/mtsi.v5i4.2025.732 (PMC12948369; doi:10.48327/mtsi.v5i4.2025.732)
Supplement: Related texts

# Research article: Identification of tick-borne borreliosis cases by qPCR from rapid malaria diagnostic tests during the Grand Magal of Touba in Senegal

## Introduction

The Grand Magal of Touba (GMT) is an annual religious event organized by the Mouride brotherhood, a Muslim community in Senegal. It takes place on the 18^th^ day of Safar, according to the Islamic calendar. It commemorates Cheikh Ahmadou Bamba Mbacke’s exile to Gabon in 1895. The GMT attracts between four and five million pilgrims, making it the largest mass gathering in West Africa. The event poses a significant risk of infectious disease transmission due to the high density of participants at the most crowded sites and the pilgrims’ diverse geographical origins [10]. A 2016 study conducted during the GMT showed that, of 20,850 patients, the most common symptoms were headaches (28.2%), followed by gastrointestinal symptoms (22%), fever (17.2%), respiratory symptoms (17.1%), and malaria (2.4%) [11]. Similarly, a study conducted from 2018 to 2021 at the Mbacké Health Center during the GMT showed that, of 141 patients, 45 (31.9%) tested positive for at least one blood pathogen: 21.3% for *Plasmodium falciparum,* 5.7% for *Borrelia* spp., and 5% for the dengue virus [5]. Of the 204 patients with fever tested for malaria with rapid diagnostic tests (RDTs), 41 (20.1%) tested positive. These RDTs were reused to screen for non-malarial pathogens, including borreliosis, through sequencing. The results revealed 18 cases (8.8%) of borreliosis, with 8.3% in non-malaria patients and 0.5% in malaria patients [7].

The goal of this study is to use PCR to identify certain pathogens that may be responsible for cases of non-malaria fever. This will be done using antigenic material extracted from RDT cassettes during the 2022 and 2023 MTAs.

## Methods

We collected ParaHIT^®^ (Akray Healthcare, India) and Bioline^TM^ Malaria Ag P.f/Pan (Abbott, USA) RDTs, which detect only *P. falciparum*, from the following health facilities in the Mbacké department: Touba Keur Niang, Darou Tanzil, Mbacké, Ndindy, Serigne Saliou, and Daroul Manane, as well as the Bagdad and Touba HLM health posts. After collecting the samples from September 13 to 17, 2022, and from September 2 to 6, 2023, we opened the RDTs, lifted the test strip, and scraped the nitrocellulose membrane, which was completely adhered to the support. Then, we transferred it to a 1.5 ml Eppendorf tube. DNA and RNA were extracted using the KingFisher™ Flex instrument. We performed all quantitative real-time PCR assays using a C1000 Touch™ thermocycler (Bio-Rad, Hercules, CA, USA) to detect *P. falciparum, P. malariae, P. vivax, P. ovale,* dengue virus (DENV), *C. burnetti, Borrelia* spp., and *Bartonella* spp. (Table I). Real-time PCR amplifications were performed using the LightCycler^®^ 480 Probes Master Kit (Roche Diagnostics, France), following the manufacturer’s instructions. Negative controls (PCR mix) and positive controls (DNA from bacterial or parasitic strains, or RNA from viral strains) were included in each series. A cycle threshold (CT) of <35 was used for positive results after amplification.

**Table I:** Primers and probes used for qPCR amplification of nucleic acids from TDR tests for malaria

Target gene	Primer name	Sequence
IS 1111 / *Coxiella burnetii*	CB_IS1111_0706F	CAAGAAACGTATCGCTGTGGC
	CB_IS1111_0706R	CACAGAGCCACCGTATGAATC
	CB_IS1111_0706P	6FAM-CCGAGTTCGAAACAATGAGGGCTG
ITS2 / *Bartonella* spp.	Bart-ITS2_F	GGGGCCGTAGCTCAGCTG
	Bart-ITS2_R	TGAATATATCTTCTCTTCACAATTTC
	Bart-ITS2_P	FAM-CGATCCCGTCCGGCTCCACCA-TAMRA
16S ribosomal RNA / *Borrelia* spp.	Bor16S-F	AGCCTTTAAAGCTTCGCTTGTAG
	Bor16S-R	GCCTCCCGTAGGAGTCTGG
	Bor16S-P	FAM-CCGGCCTGAGAGGGTGAACGG-TAMRA
*Plasmodium falciparum*	Plasmo_18S_2_MBF	AGGCAACAACAGGT CTGTGA
	Plasmo_18S_2_MBR	GCAATAATCTATCCCCATCACG
	Plasmo_18S_2_MBP	6FAM- GAACTAGGCTGCACGCGTGCTACA-TAMRA
*Plasmodium malariae*	Plasmo-mal_F	TAGCATATATTAAAATTGTTGCAG
	Plasmo-mal_R	GTTATTCCATGCTGTAGTATTCA
	Pmal_P	6FAM- TGCATGGGAATTTTGTTACTTTGAGT - TAMRA
*Plasmodium ovale*	Plasmo-ova_F	TAGCATATATTAAAATTGTTGCAG
	Plasmo-ova_R	GTTATTCCATGCTGTAGTATTCA
	Pova_P	6FAM- TGCATTCCTTATGCAAAATGTGTTC - TAMRA
*Plasmodium vivax*	Plasmo-viva_F	TAGCATATATTAAAATTGTTGCAG
	Plasmo-viva_R	GTTATTCCATGCTGTAGTATTCA
	Pviva_P	6VIC- CGACTTTGTGCGCATTTTGC - TAMRA
Virus de la dengue	Denv_F	GGA CTA GAG GTT AGA GGA GAC CCC
	Denv_R	GAG ACA GCA GGA TCT CTG GTC
	Denv_P	6FAM-AGC ATA TTG ACG CTG GGA-MGB-BHQ1

## Results

A total of 2,381 malaria rapid diagnostic tests (RDTs) were collected, including 1,014 in 2022 and 1,367 in 2023. Visual reading of the antigen tests revealed 213 positive results for *P. falciparum* (9%), including 177 (17.5%) in 2022 and 36 (2.7%) in 2023. qPCR detected *P. falciparum* in 194 samples (8.1%), including 138 (13.6%) in 2022 and 56 (4.1%) in 2023 (Table II). None of the samples tested positive for other *Plasmodium* species. Thirteen cases of tick-borne recurrent fever (TBRF) borreliosis were identified by qPCR, including one case in 2022 and twelve in 2023. Of the 13 samples that tested positive for *Borrelia,* 11 were RDT-negative and qPCR-negative for *P. falciparum;* one was RDT-negative but qPCR-posi-tive for *P. falciparum;* and one was RDT-positive and qPCR-positive for *P. falciparum.* The overall prevalence of borreliosis in RDT-negative samples was 0.5%. All samples tested negative for DENV, *C. burnetti,* and *Bartonella* spp.

**Table II:** Prevalence of different pathogens identified by qPCR from genetic material extracted from RDTs in various health facilities in the department of Mbacké, Senegal

Variables studied	Keur Niang	Darou Tanzil	Daroul Manane	Mbacké	Serigne Saliou	Health center	Bagdad	Touba HLM	Total
**2022**									
*Plasmodium falciparum*	95 (29,7%)	12 (5,4%)	5 (3,8%)	7 (5,7%)	14 (26,6%)	0	5 (7%)	0	138 (13,6%)
*Plasmodium ovale*	0	0	0	0	0	0	0	0	0
*Plasmodium vivax*	0	0	0	0	0	0	0	0	0
*Plasmodium malariae*	0	0	0	0	0	0	0	0	0
*Borrelia* spp.	0	0	0	1 (0,8%)	0	0	0	0	1 (0,1%)
*Bartonnella* spp.	0	0	0	0	0	0	0	0	0
*Coxellia Burnetti*	0	0	0	0	0	0	0	0	0
Virus de la dengue	0	0	0	0	0	0	0	0	0
**2023**									
*Plasmodium falciparum*	6 (4%)	22 (3,5%)	6 (7,5%)	3 (3,2%)	17 (6,4%)	1 (0,7%)	0	1 (14%)	56 (4,1%)
*Plasmodium ovale*	0	0	0	0	0	0	0	0	0
*Plasmodium vivax*	0	0	0	0	0	0	0	0	0
*Plasmodium malariae*	0	0	0	0	0	0	0	0	0
*Borrelia* spp.	0	3 (0,5%)	3 (3,8%)	0	4 (1,5%)	2 (1,3%)	0	0	12(0,9%)
*Bartonnella* spp.	0	0	0	0	0	0	0	0	0
*Coxellia Burnetti*	0	0	0	0	0	0	0	0	0
Virus de la dengue	0	0	0	0	0	0	0	0	0

## Discussion

Malaria caused by *Plasmodium falciparum, P. malariae,* and *P. ovale* is endemic in Senegal, and the entire population is at risk of infection [9]. Rapid diagnostic tests (RDTs) were first developed in the 1990s to facilitate malaria diagnosis in endemic areas of sub-Saharan Africa [6]. They have many advantages, including rapid results (within 5-20 minutes), ease of interpretation, and low cost. Additionally, malaria RDTs enable the rapid management of febrile patients by providing a definitive malaria diagnosis so antimalarial treatment can be administered quickly. TBRF, which is caused by *Borrelia* infections transmitted by ticks of the genus *Ornithodoros,* is a major cause of disease in several regions of Africa [2]. The geographical distribution of TBRF is linked to relatively dry climates [12]. A study conducted at the Mbacké Health Center in 2022 and 2023 showed a TBRF prevalence of 0% and 8.6%, respectively, when detected by qPCR in total venous blood from febrile patients (unpublished data). The absence of *Borrelia* infection observed in RDTs collected at the Mbacké health center in 2023 could be explained by the fact that the amount of capillary blood used for RDTs (approximately 10 pL) is much lower than that used for qPCR (200 pL of venous blood). During qPCR, amplification allows for the detection of weaker infections. These results confirm that TBRF may be present in febrile patients during the LMT and justify testing for it in non-malarial patients, preferably using whole blood.

On September 15, 2018, a dengue epidemic was diagnosed in Fatick, followed by the detection of an outbreak in Touba on October 28, 2018, where the GMT was held [1]. Of the 832 samples collected from febrile patients at various health facilities in Touba in 2018 and tested by qPCR, 22.71% (189/832) were positive for the dengue virus [3]. Studies conducted during the 2018 and 2022 GMTs showed seven cases (26.9%) and two cases (5.8%) of dengue detected by qPCR in venous blood among patients consulting for fever at the Mbacké health center [4,5]. Our study found no cases of dengue, which could be explained by the small amount of capillary blood used for rapid diagnostic tests (RDTs) or the absence of the virus at the time of the GMT.

Our study has certain limitations. Not all pathogens that can cause non-malaria fever were investigated. Clinical and demographic data, as well as the geographical origin of the patients, were not documented due to the study protocol. Additionally, the long delay between RDTs and PCR diagnosis could be reduced with better logistics.

Nevertheless, malaria RDTs used in the field are an easily accessible source of clinical samples for etiological diagnoses of fevers and for studying the epidemiology of fevers of unknown origin in GMT contexts.

## Ethics

The protocol was approved by the National Ethics Committee for Health Research in Senegal (SEN 17/62), and the study was conducted in accordance with Good Clinical Practice, as recommended by the Declaration of Helsinki and its amendments.

## Funding

This study is supported by the Institut Hospitalo-Universitaire (IHU) Méditerranée Infection and the French National Research Agency as part of the Investissements dAvenir program (reference ANR-10-IAHU-03).

## Authors’ contributions

C.D. contributed to the experimental design, wrote the manuscript, and performed the qPCR technique. I.O. contributed to the experimental design. G.D. collected samples and revised the manuscript. M.G. performed the qPCR technique. A.S., D.F., and M.F. performed the RDT dissection. P.G. drafted the original project, contributed to the experimental design, revised the manuscript, and coordinated the work. C.S. also drafted the original project, contributed to the experimental design, and coordinated the work. All authors contributed to and approved the current version of the manuscript.

## Conflicts of interest

The authors declare no conflicts of interest.
